# Understanding the Impact of Social Capital on Entrepreneurship Performance: The Moderation Effects of Opportunity Recognition and Operational Competency

**DOI:** 10.3389/fpsyg.2021.687205

**Published:** 2021-06-04

**Authors:** Gui-hua Xie, Lin-ping Wang, Bey-fen Lee

**Affiliations:** ^1^College of Economics and Management, Fujian Agriculture and Forestry University, Fuzhou, China; ^2^College of Business Administration, Fujian Business University, Fuzhou, China; ^3^Department of Hospitality Management, Chung Hwa University of Medical Technology, Tainan, Taiwan

**Keywords:** entrepreneurship performance, social capital, bonding social capital, bridging social capital, entrepreneurial ability, opportunity recognition, operational competency

## Abstract

Social capital, which is derived from psychological research, has an important value in the construction of network relationships in enterprises. It influences the direction and tendency of network connections in start-up enterprises and has gradually become an important factor in the study of entrepreneurship by scholars. However, the relationship between this and the effectiveness of innovation is unclear. In this study, the social capital is divided into bonding social capital and bridging social capital, and specific data of agricultural entrepreneurs are collected through questionnaire surveys. The results show that both bonding and bridging social capital have a significant positive effect on agricultural entrepreneurship performance. The entrepreneurial capacity of agricultural entrepreneurs regulates the relationship between social capital and creative performance. In the relationship between integrated social capital and creative performance, operational competency plays a positive role and opportunity recognition plays a negative role. On the other hand, in the relationship between bridging social capital and creative performance, the opportunity recognition plays a positive role and the operational competency plays a negative role. Finally, based on the above findings, this study proposes theoretical and practical implications and suggestions for follow-up research.

## Introduction

Social networking is important for the growth and development of both established and start-up companies, as it is an important channel for individuals, teams, and organizations to access information and resources from outside. New ventures are often born with “new entry defects” and “small size defects” because they are “new” and “small,” and thus face serious resource constraints (Siu and Bao, 2008). In implementing entrepreneurship, entrepreneurs often build and leverage social networks to access valuable information and resources, identify and develop valuable opportunities, and cultivate core competencies to create a competitive advantage and continuously ensure the sustainability of the new venture. The tendency or attitude to use the Internet to solve entrepreneurial practice problems is called as network orientation (Sorenson et al., 2008). As mentioned earlier, new ventures in China’s transitional economy face a number of constraints, a lack of resources, and a lack of credit to access the necessary resources (Long et al., 2016). As a result, they seek network relationships to solve these problems, i.e., new businesses are more network-oriented.

Watson (2007) argues that businesses are embedded in certain social relationships and their development is inevitably influenced by social relationships. When entrepreneurs or new businesses actively build network relationships and develop them for commercial behavior, they exhibit a strong network orientation, which satisfies the basis for building and maintaining a competitive advantage (Strobl and Kronenberg, 2016; Mu et al., 2017). Adler and Kwon (2002) suggest that the social capital theory refers to the existence of social networks in which individuals establish special social relationships through their interactions with others, and the resources or information that actors obtain through these interactions. Through close social interaction, the efficiency, depth, and breadth of knowledge exchange between individuals is increased (Lane and Lubatkin, 1998). According to Sorenson and Stuart (2008), the ability of new enterprises to face changes in the industry environment and grasp business opportunities in the process of operation is a test of whether they can improve the quality and quantity of information they obtain in an open environment and have good thinking, innovation, and responsiveness. If companies can quickly recognize changes in the market and react and act quickly in their operation management, the competitive advantage they build will be enhanced and sustained (Chen, 2019). Therefore, if we can grasp the advantageous structural capital of networking, together with the common cognitive network and the shared trust relationship, we can not only stimulate each other’s all-round learning but also increase the knowledge exchange frequency, so the willingness and ability of organization members to share knowledge could be enhanced. In addition, the social capital of reciprocal interest combination has the effect of stimulating the connection and exchange of resources in the organization, which can accelerate the expansion and circulation of knowledge.

At present, many national policies encourage the development of agricultural and rural innovation and entrepreneurship to promote the revitalization of rural areas. Social capital is a special and important asset for agricultural entrepreneurs and has a positive effect on entrepreneurial activities in rural areas and has been widely studied in studies of agricultural entrepreneurship performance. Scholars have explored the impact of differential social capital, such as geographic, kinship, and karma social capital, on entrepreneurship (Lans et al., 2015). With the development of technology and changes in the economic structure of rural societies, social capital for agricultural entrepreneurs has also changed, with more sources and a wider variety (Rezaei-Moghaddam and Izadi, 2019). These social capitals can be divided into bonding social capital, which exhibits strong ties, and bridging social capital, which exhibits weak ties (Leonard, 2004; Newell et al., 2004; Agnitsch et al., 2006; Coffé and Geys, 2007; Jensen and Jetten, 2015). However, there is a lack of research on these two types of social capital of entrepreneurs and their relationship with entrepreneurial performance in the field of agricultural entrepreneurship. Therefore, this study focuses on the impact of bonding and bridging social capital on agricultural entrepreneurial performance with agricultural entrepreneurs as the research object and uses two different types of entrepreneurial abilities, namely, opportunity recognition and operational competency, as moderating variables to further analyze the role of resources and entrepreneurial factors on agricultural entrepreneurial performance.

## Literature Review

### Social Capital and Entrepreneurial Performance

Social capital was first introduced by sociologists to explain the use of general interpersonal embedded relationships, such as community, friends, colleagues, and family relationships, to help to create social capital and wealth for individuals (Burt, 1997). Jacobs (2016) defines social capital as an interpersonal relationship that is cultivated over time and that provides a good foundation for group trust, cooperation, and collective action. The social capital theory focuses on how interpersonal relationships cultivated over time can provide a valuable resource for the members of a network.

Social capital is a collection of the most important resources for entrepreneurs including social capital at the individual and social levels. This study focuses on the social capital of entrepreneurs at the individual level. Social capital was first described by Bourdieu (1977), who defined it as “the sum of actual or potential resources associated with an enduring network of more or less institutionalized relationships of mutual understanding and recognition. From a functional perspective, social resources are social capital (Coleman, 1988). Lin (2001) divides social resources into personal resources and social resources. In his view, social resources are embedded in a network of personal relationships and originate from an individual’s interpersonal relationships, and only when an individual interacts with other members of society, social resources are generated. Based on this, he proposed the social capital theory, defining social capital as “the social resources that exist in social network relationships and can bring returns”. This study draws on the research by Lin (2001) to define the social capital of entrepreneurs as the various networks of relationships and the resulting social resources of entrepreneurs in the process of starting a business.

Two sources consist of social capital for entrepreneurs: internal networks and the resources they bring with them, which have strong relational characteristics, called as bonding social capital; and external networks and relationships, and the resources embedded in them, which have weak relational characteristics, called as bridging social capital (Sajuria et al., 2015; Ceci et al., 2019). The former provides emotional support through internal interactions, shares information, and promotes trust among internal members; the latter facilitates entrepreneurs to obtain information from outside the organization, identify opportunities, and gain decision-making advantages.

This paper draws on Putnam (2000) and Phua et al. (2017) to classify social capital in social media contexts into bonding social capital and bridging social capital. Among them, bonded social capital refers to the scope of interaction, frequency of interaction, degree of trust, and reciprocity formed by the network of communication and interaction between entrepreneurs and their familiar friends and relatives as well as within the entrepreneurial team through social media. Bridging social capital refers to the social capital formed by entrepreneurs with the help of social media and different relationship networks of suppliers, customers, the public, government departments, service organizations, media, and intermediaries in the external environment, including the breadth of relationships, the depth of relationships, the degree of trust in relationships, and the degree of reciprocity between relationships.

Organizational behavior scholars believe that entrepreneurial performance, or entrepreneurial organizational performance, is a measure of how well an entrepreneurial organization accomplishes its goals and is often used to measure the outcomes and effectiveness of entrepreneurship (Lumpkin and Dess, 2001; Hmieleski and Corbett, 2008; Renko et al., 2015). Morgan et al. (2010) argue that the performance and benefits of farmers’ farming-related entrepreneurship can be measured by comparing the performance of farmers before and after starting a business or by comparing the benefits of similar entrepreneurs. The definition of entrepreneurial performance in this study is the results obtained and the extent to which the entrepreneurs has achieved his or her goals after starting a farm-related business.

### The Impact of Social Capital on the Performance of Entrepreneurship

Scholars have studied the relationship between social capital and entrepreneurship and found that the social capital of entrepreneurs facilitates the adoption of entrepreneurial behaviors by entrepreneurs (Wang et al., 2019). For example, Li et al. (2021) empirically showed that social capital not only directly promotes farmers’ e-commerce adoption but also plays a part in the positive relationship between “prior entrepreneurial experience-farmers’ e-commerce adoption behavior” and “prior training experience-farmers’ e-commerce adoption behavior.” An empirical study by Kobayashi et al. (2006) found that in the e-commerce environment, rural residents gained a heterogeneous and broader social network, reduced affective trust dependence, adapted to market social norms, and developed new and richer social capital, which ultimately facilitated entrepreneurial activities. Therefore, social capital for entrepreneurs is conducive to the innovation and growth of entrepreneurial enterprises and ultimately to the improvement of entrepreneurial performance. The relationship between integrated social capital and bridging social capital and entrepreneurial performance is addressed.

Empirical research has found that bonding social capital can provide information on value creation capabilities (Herrero, 2018). Family, relatives, or friends with whom the entrepreneur interacts regularly provide information on raw materials, capital, pipelines, and internal production management (Wernerfelt, 1984; Davidsson and Honig, 2003). The bonding social capital formed among familiar members can facilitate the entrepreneurial activities of entrepreneurs in terms of resource provision, emotional support, and psychological enhancement. First, acquaintances or family members provide a source of capital to start a business and make up for the lack of entrepreneurial labor. The start-up capital for small and micro-agricultural entrepreneurial activities in China comes from family members or family capital, and the labor force at the early stage of entrepreneurship is mainly family members, and some micro-entrepreneurs even have only their own people involved. Secondly, the entrepreneurial process is full of hardships and the understanding of family members, relatives, and friends as well as the entrepreneurial team members often serves as a spiritual pillar for the entrepreneur during difficult times, strengthening the entrepreneur’s resilience and making him less likely to give up. In summary, it is concluded that

*Hypothesis* 1: The bonding social capital of entrepreneurs has a significant positive effect on the performance of entrepreneurship.

Previous research has shown that bridging social capital has a positive effect on entrepreneurial performance, specifically in terms of entrepreneurial heterogeneity in resource acquisition, identification and acquisition of entrepreneurial opportunities, and innovative business ideas (Stam et al., 2014; Lee et al., 2019). Bridging social capital can provide entrepreneurs with heterogeneous information about the market (Stam et al., 2014). Entrepreneurs receive entrepreneurial guidance through various channels, informal relationships with people inside and outside the industry, and participation in professional discussions can facilitate the recognition of entrepreneurial opportunities (Spigel, 2017).

In agricultural entrepreneurship, entrepreneurial projects are mostly scattered in large areas of the countryside, and some entrepreneurial activities are carried out in remote rural areas. As a micro and small business start-up, agricultural entrepreneurs themselves often have to personally participate in agricultural production and operation activities and cannot spend too much time and energy to carry out social capital operations. Therefore, in the old closed rural environment, the social capital of farmers was mainly the traditional social capital based on blood, kinship, and locality, and such social capital had few opportunities to obtain heterogeneous resources because they were familiar with each other. In this context, those who have access to more favorable heterogeneous resources in traditional rural societies are often agricultural entrepreneurs whose family members are civil servants in government departments, and they have better entrepreneurial performance because of the heterogeneity of government and business relationship resources. In summary, the second research hypothesis of this study was derived.

*Hypothesis* 2: The bridging social capital of entrepreneurs has a significant positive impact on the performance of entrepreneurship.

### Entrepreneurial Ability and Its Moderating Effect

#### Entrepreneurial Ability

As an important quality for entrepreneurs, entrepreneurial ability has received widespread attention from academics. In the past, scholars considered as entrepreneurial competencies as qualities and skills necessary for entrepreneurs to carry out the whole process of entrepreneurial activities (Pyysiäinen et al., 2006; Chell, 2013). McElwee and Bosworth (2010) defined family farmer entrepreneurial competencies as those that family farmers should possess to identify and develop family farm entrepreneurial opportunities, obtain the resources needed to start a farm, and implement entrepreneurial activities. In summary, this study considers that the entrepreneurial capacity of agricultural entrepreneurs refers to the various qualities and abilities of agricultural entrepreneurs to give full play to their initiative, identify and develop opportunities for agriculture-related entrepreneurship, and carry out decision-making, resource utilization, and organizational management for the normal operation and management of agricultural projects.

In this study, the entrepreneurial ability of entrepreneurs is divided into two dimensions: opportunity recognition and operational competency. Gatewood et al. (2002) suggest that new firms should identify and develop opportunities and use them to build organizational capabilities to achieve business growth defined as opportunity recognitions. In this study, opportunity recognition refers to the ability of entrepreneurs to identify, through effective information obtained in the process of entrepreneurship, development opportunities that are favorable to their own operations, such as new products and markets with development prospects, and to put these opportunities into practice in entrepreneurship.

Regarding operations management capability, Burke et al. (2002) argue that it is the ability to build and grow an organization and is a timely response that reflects the effectiveness of an organization’s operations management process. Alsos et al. (2003) identified operational management capability as part of entrepreneurial capability and the ability of farmers to coordinate and integrate entrepreneurial resources after implementing entrepreneurial activities, to make the best use of existing conditions to operate and manage new ventures, and to strive to improve entrepreneurial performance. It specifically refers to the ability of entrepreneurs to integrate various resources in their entrepreneurial activities, manage the production and services of entrepreneurial activities internally, motivate the leadership of the team, develop social networks externally, communicate and link up, continuously solve various problems that arise in the process of entrepreneurship, and ultimately achieve the expected results of entrepreneurship.

#### Moderating Effect of Entrepreneurial Ability

Past research has validated the moderating role of entrepreneurial capacity (Huang, 2016). Opportunity recognition can mediate the relationship between network orientation and the competitive advantage of new businesses. The effect of network concern and openness on competitive advantage is more pronounced for new firms with strong opportunity recognition, while the effect of network cooperativeness on competitive advantage is suppressed (Reed et al., 2012). Past empirical studies have found that the relationship between business models of start-ups and organizational performance is positively influenced by entrepreneurial capabilities (Cucculelli and Bettinelli, 2015). In addition, it has been shown that this positive moderating effect of entrepreneurial competencies also occurs between entrepreneurial relationship network construction and organizational performance relationships (Zahra and Garvis, 2000; Stam et al., 2014).

In entrepreneurial activities, consumer demand for products and services changes rapidly, requiring entrepreneurs to have the ability to dynamically grasp entrepreneurial opportunities, i.e., to both identify opportunities and make full use of them. With the improvement of opportunity recognition, the more entrepreneurs can discover favorable business opportunities through different social capital, including new products popular in the market, innovative business services, and occupy the market at favorable times, thus obtaining better financial performance, innovation performance, and customer satisfaction. Summarizing the above analysis, it is concluded that

*Hypothesis* 3a: The opportunity recognition of entrepreneurs significantly and positively moderates the positive effect of bonding social capital on entrepreneurial performance.

*Hypothesis* 3b: The opportunity recognition of entrepreneurs significantly and positively moderates the positive effect of bridging social capital on entrepreneurial performance.

In addition to the opportunity recognition, the operations ability to effectively integrate and organize various resources is also important (Camisón and Villar-López, 2014). A strong operational competency enables the allocation of existing resources to products and services that will enable customers’ needs to be more fully met or will enable the business to meet customers’ needs at a lower price. In business, the combination of social capital and bridging social capital by entrepreneurs brings rich entrepreneurial resources to the business activities of projects. Once operational competency are in place, these social capital will enable entrepreneurial ventures to be more responsive and flexible in terms of improving quality, reducing costs, and innovating operations (Coltman and Devinney, 2013). With the improvement of operational competency, the positive impact of social capital on the entrepreneurial performance will become stronger and stronger. In summary, the above studies have resulted in

*Hypothesis* 4a: The operational competency of entrepreneurs significantly and positively moderates the positive effect of bonding social capital on the entrepreneurial performance.

*Hypothesis* 4b: The operational competency of entrepreneurs significantly and positively moderates the positive effect of bridging social capital on the entrepreneurial performance.

**Figure 1:**
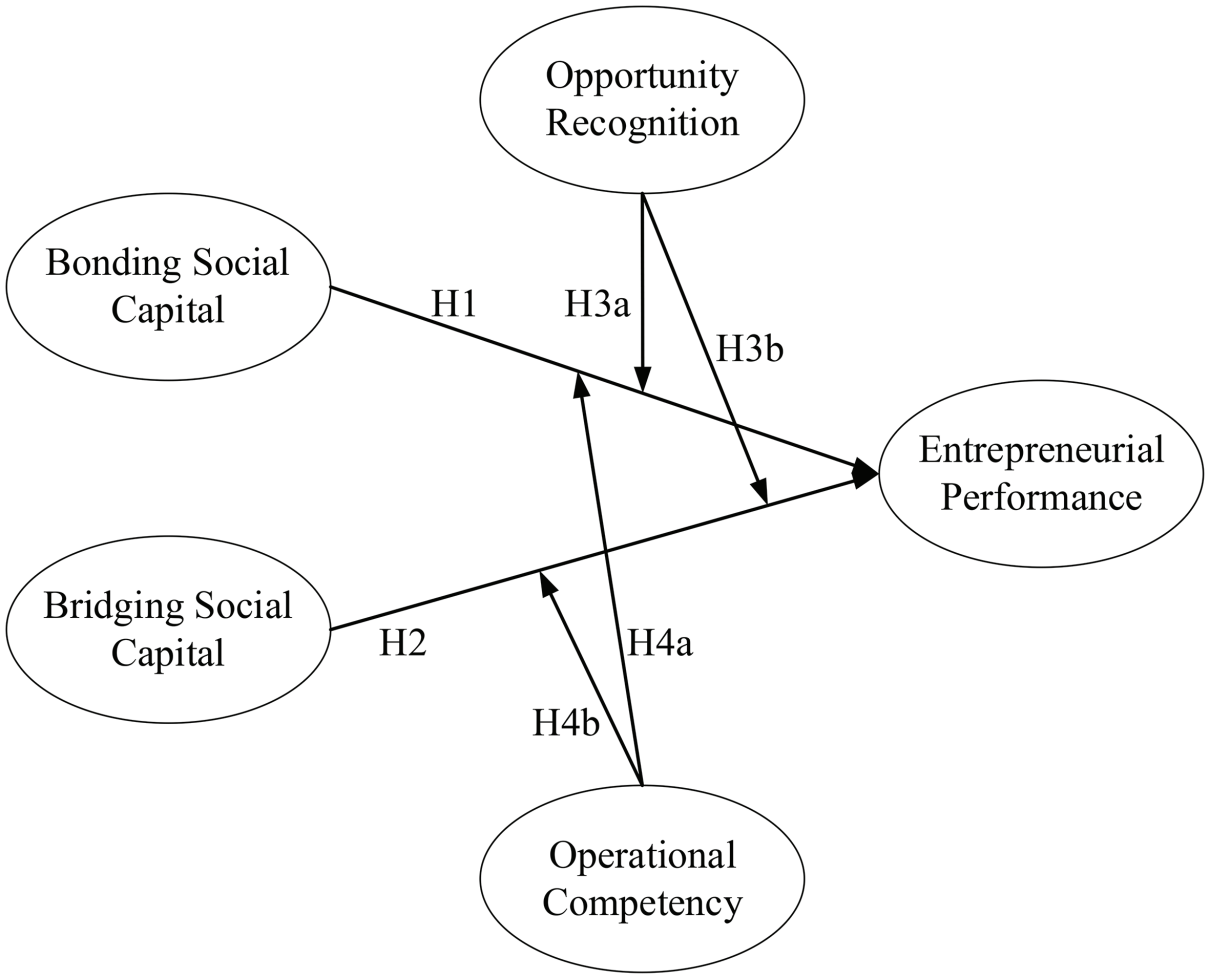
Research model.

Based on the literature review and the hypothesis proposed, this study proposes the following research model for Figure 1.

## Research Methods

In this study, the social capital of entrepreneurs was classified into bonding social capital and bridging social capital, and the questionnaire was referred to the scales of Subramaniam and Youndt (2005) and Han and Hovav (2013). The measure of entrepreneurial performance was subjective and was based on the scale of Su et al. (2015). We employed the following steps to select scale items. First, the scale items from the prior literature were translated into Chinese. Second, three professors familiar with social capital and agricultural contexts issues in China were asked to examine the Chinese wording of each measurement item and suggest on its content validity. These suggestions were adapted to add, remove, or reword inappropriate scales. Third, the pilot test was conducted prior to the formal investigation to enhance the readability and clarity of all the measurement items. The questionnaire in this study was scored on a 7-point Likert scale. The target of this paper is startup enterprises, but there is no consensus among scholars on the criteria for new enterprises. According to Zahra and Bogner (2000), firms that have been in existence for less than 8 years are start-up enterprisers. Second, in this study, 180 respondents were distributed for pre-test, 166 were returned, and 120 valid questionnaires remained after questionnaire screening. The reliability of the data was examined by internal consistency reliability analysis. The data were subjected to internal consistency reliability analysis for reliability check, and the Cronbach’s *α* values for each dimension ranged from 0.74 to 0.82, which were higher than the reliability standard of 0.7, indicating that the questionnaire was reliable. The measurement items of social capital scale, the entrepreneurial performance scale, and the entrepreneurial ability scale have KMO values greater than 0.7, the Bartlett sphericity test significance is less than 0.05, and the factor loading of all measured questions is greater than 0.6, which meets the default criteria. Therefore, the scale of this study has good construct validity.

The formal survey of this study, which began in July 2018 and continued through March 2019, was conducted on agricultural entrepreneurs in China and new vocational farmer training classes at agricultural vocational and technical colleges. A total of 326 paper and electronic questionnaires were distributed in this study, and 308 valid questionnaires were analyzed using SPSS and AMOS software after excluding invalid questionnaires. As can be seen from Table 1, among the agricultural entrepreneurs in this survey, there are 155 men, accounting for 50.3%, mainly under the age of 40 (89.3%), educated mainly in high school (or secondary school; 52.9%), nearly 80% of the entrepreneurs are married, and the number of those engaged in large-scale agricultural farming or breeding is the highest, reaching 28.6%. The majority of entrepreneurs operate as small and medium-sized individual households, family farms, and large agricultural households, and the team size is generally small, with 59.7% up to 10 people, and 80.8% of the entrepreneurial projects have been operating for less than 5 years.

**Table 1:** Basic description of the official survey sample.

**Item**	**Category**	**Frequency**	**Percentage**	**Item**	**Category**	**Frequency**	**Percentage**
Gender	Male	155	50.3	Operation form	Family farm	66	21.4
	Female	153	49.7		Large breeders	67	21.8
Age	Under 30 years old	140	45.5		Cooperatives	50	16.2
	31–40 years old	135	43.8		Agricultural company	36	11.7
	41–50 years old	24	7.8		Small- and medium-sized individual operators	89	28.9
	Over 51 years old	9	2.9	Team size	Up to 10 people (inclusive)	153	49.7
Education	Elementary school and below	4	1.3		11–30 people	99	32.1
	Junior high school	44	14.3		31–50 people	35	11.4
	High school (or middle school)	163	52.9		51 people (or more)	21	6.8
	University	95	30.8	Operating time	Less than (including) 1 year	39	12.7
	Graduate students	2	0.6		2–3 years	123	39.9
Marriage	Unmarried	65	21.1		4–5 years	87	28.2
	Married with children	36	11.7		6–7 years	30	9.7
	Married with no children	207	67.2		8–9 years	12	3.9
Area of interest	Large-scale planting or cultivation	88	28.6		More than 10 years	17	5.5
	Distribution of agricultural materials (fertilizers, seeds, agricultural tools, etc.)	43	14.0	Distance from town	Less than 10 km (inclusive)	109	35.4
	Agricultural products processing	39	12.7		11–30 km	137	44.5
	Agricultural products sales	77	25.0		31–50 km	46	14.9
	Leisure agriculture and rural tourism	27	8.8		51 km (inclusive) or more	16	5.2
	Other agriculture-related industries and services	34	11.0				

## Result

### Reliability and Validity Analysis

This study evaluates and revises the CFA measurement model based on the two-stage model. The results of the analysis of the measurement model using the extreme likelihood estimation method are shown in Table 2. The standardized factor negative loadings ranged from 0.611 to 0.822, all of which met the range, indicating that each question had question reliability; the synthetic reliability of the study constructs ranged from 0.838 to 0.893, all of which exceeded 0.7, all of which met the criteria suggested by scholars, indicating that each construct had good internal consistency; finally, the mean variance extractions ranged from 0.510 to 0.602, all of which were above 0.5, indicating that each construct had good internal consistency. Finally, the mean variance extracted ranged from 0.510 to 0.602, all above 0.5, all of which met the criteria of (Hair et al., 2017). The average variance extracted (AVE) comparison method was used to examine the discriminant validity of the measurement model. The square root of AVE for each variable is greater than the correlation coefficient between the variables, which meets the criteria of Fornell and Larcker, indicating a good discriminant validity between the variables.

**Table 2:** Analysis of measurement model results.

**Construct**	**Indicator**	**Mean (SD)**	**Standardized factor loading**	**Composite reliability**	Average variance extracted (**AVE)**
Bridging social capital (BRSC)	BRSC1	5.56 (1.055)	0.781	0.883	0.602
	BRSC2	5.45 (1.101)	0.802		
	BRSC3	5.35 (1.184)	0.733		
	BRSC4	5.38 (1.182)	0.745		
	BRSC5	5.40 (1.139)	0.815		
Bonding social capital (BOSC)	BOSC1	5.63 (1.133)	0.769	0.893	0.582
	BOSC2	5.64 (1.023)	0.777		
	BOSC3	5.70 (1.134)	0.705		
	BOSC4	5.48 (1.029)	0.822		
	BOSC5	5.40 (1.046)	0.785		
	BOSC6	5.53 (1.090)	0.712		
Entrepreneurship performance (EP)	EP1	5.72 (0.873)	0.673	0.867	0.566
	EP2	5.36 (1.106)	0.762		
	EP3	5.17 (1.151)	0.721		
	EP4	4.97 (1.321)	0.817		
	EP5	5.33 (1.195)	0.780		
Opportunity recognition (OR)	OR1	4.95 (1.325)	0.665	0.849	0.585
	OR2	5.02 (1.349)	0.757		
	OR3	4.90 (1.389)	0.821		
	OR4	4.97 (1.363)	0.807		
Operational competency (OC)	OC1	5.40 (1.124)	0.747	0.838	0.510
	OC2	5.43 (1.017)	0.762		
	OC3	5.37 (1.124)	0.752		
	OC4	5.42 (1.087)	0.688		
	OC5	5.83 (1.028)	0.611		

Comparing the square root of the AVE of a given construct with the correlations between the construct and the other constructs is the discriminant validity (Fornell and Larcker, 1981). The indicators are more closely related to the construct than the others if the square root of the AVE of a construct is greater than the off-diagonal elements in the corresponding rows and columns.

As shown in Table 3, the bold numbers in the diagonal direction represent the square roots of AVEs. Because the square roots of AVEs in the diagonal direction are all greater than the off-diagonal numbers, discriminant validity is satisfactory for all constructs.

**Table 3:** Discriminant validity for the measurement model.

	**AVE**	**BOSC**	**BRSC**	**EP**	**CA**	**OA**
BOSC	0.582	**0.763**				
BRSC	0.602	0.577	**0.776**			
EP	0.566	0.509	0.494	**0.752**		
OR	0.585	0.479	0.521	0.741	**0.765**	
OC	0.510	0.541	0.564	0.678	0.707	**0.714**

BRSC, bridging social capital; BOSC, bonding social capital; EP, entrepreneurship performance; OR, opportunity recognition; and OC, operational competency. The items on the diagonal on bold represent the square roots of the AVE; off-diagonal elements are the correlation estimates.

The measurement model analysis was performed by the maximum likelihood estimation method. After the correction of the cardinality heteroskedasticity, all the fitted indicators improved significantly and the model fit was adequate as shown in Table 4. The goodness-of-fits of the model shows the model meets the criteria, indicating that the model has good fit indices.

**Table 4:** Model fit.

**Fit index**	**Allowable range**	**Model fit**	**Adjusted Model fit**
Chi-square test	The smaller the better	256.671	171.021
Degree of freedom	The bigger the better	101.000	101.000
Chi-square test/degree of freedom	Greater than 1 and less than 3	2.541	1.693
Root mean square error of approximation	<0.08	0.071	0.047
Standardized RMR	<0.08	0.042	0.042
Tucker-Lewis index (Non-normed Fit Index)	>0.9	0.931	0.953
Comparative Fit Index	>0.9	0.942	0.961
Fitting Optimization Index	>0.9	0.909	0.939
Adjusted Fitting Optimization Index	>0.9	0.892	0.928

### Structural Model Analysis

From the results of the path coefficient in Table 5, bridging social capital (*b* = 0.214, *p* < 0.001) and bonding social capital (*b* = 0.228, *p* < 0.001) significantly affect entrepreneurial performance, and research Hypothesis 1 and research Hypothesis 2 hold, and the explanatory power of bridging social capital and bonding social capital in explaining entrepreneurial performance is 32.0%. It can be seen that social capital, which is rich in social relationships and social resources, has a significant and important impact on entrepreneurial performance for agricultural entrepreneurs.

**Table 5:** Structural model results.

**Dependent variable**	**Independent variable**	**Unstandardized regression coefficients**	**Standard error**	**T-value**	***p***-value	**Standardized Regression Coefficients**	**Explained variance**
Entrepreneurship performance	Bonding social capital	0.231^***^	0.055	3.823	0.000	0.293	0.323
	Bridging social capital	0.231^***^	0.052	4.426	0.000	0.342	

Age (control variable 1): Beta = −0.020, *p* > 0.05; Education (control variable 2): Beta = −0.012, *p* > 0.05; Operation form (control variable 3): Beta = 0.016, *p* > 0.05; Team size (control variable 4): Beta = −0.010, *p* > 0.05.

*** *p* < 0.001.

### Analysis of Moderation Effect

In this study model, opportunity recognition and operational competency are the moderators. As shown in Table 6, in terms of opportunity capacity, the moderation effect of bonding social capital*opportunity capacity on entrepreneurial performance is −0.090 (*t* = |−0.503| < 1.96, *p* = 0.615 > 0.05), which means that the moderation effect does not exists. The research Hypothesis 3a is not supported. The moderating effect of bridging social capital*opportunity capacity on entrepreneurial performance is 0.512 (*t* = |2.042| > 1.96, *p* = 0.041 < 0.05), indicating that the moderating effect exists and that the slope of bridging social capital on entrepreneurial performance increases by 0.512 units for each unit increase in the moderating variable opportunity capacity, and research Hypothesis 3b is supported. This may be because bridging social capital is the sum of social network relationships based on weak ties and the heterogeneous resources they can bring, which can bring more differentiated resources and means more opportunities. Therefore, the stronger the ability of agricultural entrepreneurs to identify and utilize opportunities, the more they tend to look for better entrepreneurial and innovative development opportunities from weak ties rich in bridging social capital, thus bringing better creative performance to agricultural business activities.

**Table 6:** Analysis of the moderation effect.

**Variable**	**Un value**	**Standard error**		**T-value**	***p***-value
Bonding social capital*opportunity recognition	−0.090^*^	0.179		−0.503	0.615
Bridging social capital*opportunity recognition	0.512^*^	0.251		2.042	0.041
Bonding social capital*operation competency	0.724^**^	0.276		2.628	0.009
Bridging social capital*operation competency	−0.230^*^	0.175		−1.315	0.188

* *p* < 0.05;

** *p* < 0.01.

In terms of operating capacity, the moderation effect of bonding social capital*operation competency on entrepreneurial performance is 0.724 (*t* = |2.628| > 1.96, *p* = 0.009 < 0.05), indicating the existence of a moderation effect, representing that for every unit increase in the moderation variable operating capacity, the slope of bonding social capital on entrepreneurial performance increases by 0.724 units, and research Hypothesis 4a is supported. The moderation effect of bridging social capital*operation competency on entrepreneurial performance is −0.230 (*t* = |−1.315| < 1.96, *p* = 0.188 > 0.05), indicating that the moderation effect does not exist significantly, and research Hypothesis 4b is not supported. This is probably because the operational competency is more a reflection of the entrepreneur’s ability to internally coordinate and manage the business project. The stronger the operational competency of agricultural entrepreneurs, the more they will pay attention to the development of strong relationships for agricultural business projects and will focus on existing network relationships and resources to improve the survival and development of agricultural business projects through their integrated operations.

## Conclusion

New enterprises often have inherent new entry defects and small-scale defects because they are new and small, and thus face serious resource constraints. Therefore, social capital is important for the growth and development of both established and new businesses, as it is an important channel for individuals, teams, and organizations to obtain information and resources from outside sources (Zhou et al., 2020). In the process of implementing entrepreneurship, entrepreneurs typically build and leverage social capital to acquire valuable information and resources, identify and develop valuable opportunities, and cultivate core competencies to create a competitive advantage and continuously ensure the sustainability of the new venture.

The following conclusions were drawn from an empirical study of 308 agricultural entrepreneurs: (1) both bonding social capital and bridging social capital significantly and positively affect the performance of agricultural entrepreneurship and (2) there are differences in the way agricultural entrepreneurs with different abilities use social capital to start entrepreneurial activities and their effectiveness. The use of bridging social capital by entrepreneurs with strong opportunity capabilities will significantly contribute to the improvement of entrepreneurial performance, while entrepreneurs with strong operational competency will achieve better results by using bonding social capital to start agricultural entrepreneurship.

In response to the above findings, the following recommendations were proposed. First, entrepreneurship should win the support of relatives and stakeholders obtain more bonding social capital; on the other hand, it should also obtain heterogeneous resources and accumulate bridging social capital to eventually promote the smooth development of entrepreneurial activities. Second, entrepreneurs objectively understand the differences in their own abilities and make full use of their strengths to carry out entrepreneurial activities. The members of the entrepreneurial team can reasonably divide the work and form complementary capabilities in terms of opportunity capabilities and operational competency, thus promoting the overall improvement of organizational performance.

This study enriches the study of factors influencing the entrepreneurial performance of agricultural entrepreneurs, provides a new idea for the study of social capital of agricultural entrepreneurs, and once again verifies the important role of entrepreneurial ability of entrepreneurs at the individual level in regulating entrepreneurial performance. Social capital is only one of the factors influencing entrepreneurship performance. Future research can combine other factors to conduct a comprehensive analysis of the impact on entrepreneurship performance or to consider the mediating factors of social capital of agricultural entrepreneurs on entrepreneurship performance and reveal the specific path of its role.

Finally, this study has three main research limitations and future research directions. First, since this study focuses on cross-sectional analysis, it cannot be interpreted for a specific period of time. In the future, we may use time series or longitudinal analysis to investigate the relationship between social capital, entrepreneurial performance, and national competitiveness over time with comparing the results of this study. Second, this article uses structural equation modeling as the main statistical analysis method. Structural equation modeling is a statistical methodology of parametric estimation, which aims to use the characteristics of the sample inference matrix. To avoid bias in statistical inference, the data collection must conform to the assumptions of sampling principles. For example, the structural equation model is estimated with the assumption of simple random sampling, i.e., any sampling unit in the parent has an equal chance of being selected as a sample. Due to the difficulty of obtaining a sample list for this study, intentional sampling was adopted for data collection. Therefore, the statistical inferences obtained from the theoretical model can only be generalized to matrices that are similar with the observed samples in this study, but not to general matrices. Third, the selection of relevant factors in this study did not compare the differences in social culture, religion, economic income, and geographical location. In other words, future studies may adopt different analytical frameworks, including Western and Eastern cultures, high-income and low-income countries, advanced countries and developing countries, etc., and use different frameworks as control variables to investigate the differences in social capital among different groups and their effects on entrepreneurial performance and national competitiveness.

## Data Availability Statement

The raw data supporting the conclusions of this article will be made available by the authors, without undue reservation.

## Ethics Statement

Ethical review and approval was not required for the study on human participants in accordance with the local legislation and institutional requirements. Written informed consent for participation was not required for this study in accordance with the national legislation and the institutional requirements.

## Author Contributions

G-hX and L-pW: conceptualization. G-hX: methodology investigation, data curation, and writing – original draft preparation. G-hX, L-pW, and B-fL: validation. B-fL: formal analysis and writing – review and editing. L-pW: supervision and funding acquisition. All authors have read and agreed to the published version of the manuscript.

### Conflict of Interest

The authors declare that the research was conducted in the absence of any commercial or financial relationships that could be construed as a potential conflict of interest.
